# Extra-abdominal growth of a large low-grade appendiceal mucinous tumour through the femoral canal–a rare case report

**DOI:** 10.3389/fonc.2024.1396265

**Published:** 2024-06-11

**Authors:** Yan Kang, Junfeng Ma, Xiaolong Li, Zhong Yang, Mingxu Da

**Affiliations:** ^1^ The Frist School of Clinical Medicine, Gansu University of Traditional Chinese Medicine, Lanzhou, China; ^2^ Department of Surgical Oncology, Gansu Provincial Hospital, Lanzhou, China

**Keywords:** pseudomyxoma peritonei, cytoreductive surgery, hyperthermic intraperitoneal chemotherapy, oncology, low-grade appendiceal mucinous neoplasms

## Abstract

Low-grade appendiceal mucinous neoplasms (LAMNs) are rare and heterogeneous diseases that, despite their increased incidence, are well differentiated, tend to be painless, and histologically lack distinctive invasive features without infiltrative growth, destructive infiltration, or associated pro-fibroproliferative responses. However, the biological behaviour of these tumours is difficult to determine preoperatively or intraoperatively, and the possibility of rupture puts patients at risk for peritoneal pseudomucinous neoplasms (PMPs).Patients with low-grade appendiceal mucinous tumours and peritoneal pseudomucinous tumours experience slow disease progression and are incurable and have a high risk of recurrence, morbidity, and ultimately death, despite the reported 5- and 10-year survival rates of 50–86% and 45–68%, respectively. In this article, we report the case of a 80-year-old male with a giant low-grade appendiceal mucinous tumour associated with a peritoneal pseudomucinous tumour, and discuss the diagnostic and management strategies for giant low-grade appendiceal mucinous tumours in the context of a literature review.

## Introduction

Appendiceal mucinous neoplasms (AMNs) are rare tumours that account for less than 1% of all cancers. The treatment of AMNs is not well defined, and controversy exists regarding the extent of surgery and the role of chemotherapy, including early postoperative intraperitoneal chemotherapy (EPIC) and hot intraperitoneal chemotherapy (HIPEC). Peritoneal pseudomucinous tumours (PMP) are diffuse aggregates of gel-like material in the abdomen and pelvis, as well as mucinous implants on the peritoneal surface. Follow-up studies have shown that most PMPs arise from the appendix and represent localised spread into the peritoneal cavity (1).Here, we report the case of a giant low-grade appendiceal mucinous tumour associated with a peritoneal pseudomucinous tumour.

## Case description

**Figure 1:**
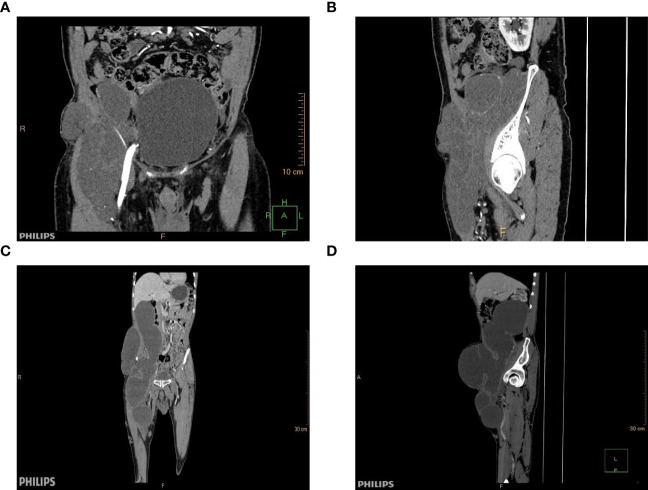
Contrast-enhanced CT image before and after three years. **(A)** Coronal contrast-enhanced CT image obtained in 2020. **(B)** 2020 contrast-enhanced CT image of a dislocation. **(C)** Coronal contrast-enhanced CT image obtained in 2023. **(D)** Contrast-enhanced CT image of a dislocation.

An 80-year-old male patient presented to our clinic due to a large palpable mass in the right inguinal ligament, exacerbating pain during activity and preventing independent ambulation for the past two months. Three decades prior, the patient had been diagnosed with a right inguinal mass, approximately 2cm in size. He did not pursue treatment due to lack of concern. This patient previously sought medical attention for “cerebral infarction” three years ago, and a pelvic CT scan revealed multiple, solitary, cystic lesions in the right quadriceps femoris and right obturator internus muscle, spanning an 8.5cmx10.1cmx19.7cm range. At that time, the doctor recommended surgical intervention, but the patient refused and asked to be discharged. On examination, a large palpable mass was palpable in the right ilium, right inguinal region, and right lower extremity femur, in the form of an irregular shape. The mass extended from the umbilical region to the iliac crest, and from the mid-shaft of the femur to the abdomen. A pelvic CT scan indicated a giant, multi-cystic tumour in the liver, right ilium, and right femur, spanning a 14.6cmx17.5cmx40.5cm range. The tumour markers showed AFP (alpha-fetoprotein) of 1.48 ng/mL, CEA (carcinoembryonic antigen) of 41.98 ng/mL, and CA125 and CA199 were not significantly abnormal (**Figure 1**).

**Figure 2:**
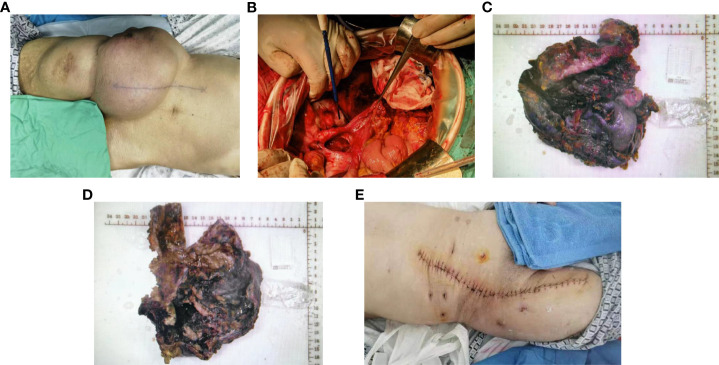
Excision of the appendix and periappendiceal mass, right iliofoliac lumbar mass resection, right subinguinal and suprafemoral mass resection. **(A)** Surgical area. **(B)** Excision of the appendix and periappendiceal masses. **(C-D)** The overall characteristics of the sample. **(E)** Postoperative incision healing.

**Figure 3:**
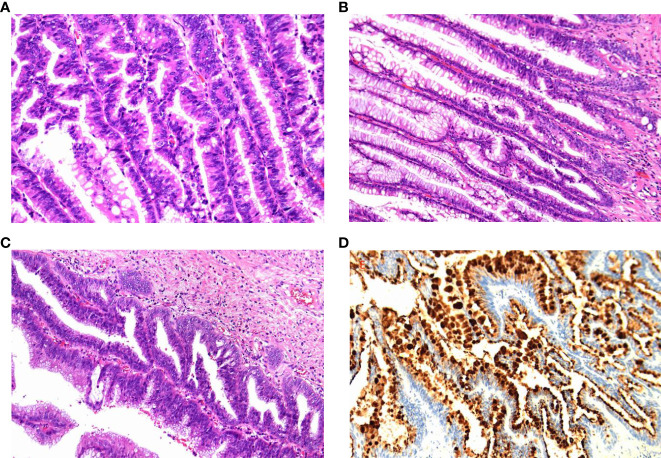
Pathological findings. **(A)** Low-grade mucinous tumours of the appendix have incomplete appendix muscles, and the tumour penetrates the visceral peritoneum and involves the striated muscle tissue of the abdominal wall. **(B)** (Right inguinal skeletal muscle mass, right abdominal wall mass) A large amount of mucus or gelatinous material was measured in the fibrous fat, and a low-grade mucinous tumour of the peritoneum (pseudomyxoma peritonei) was considered. **(C)** (Abdominal wall lesion) A low-grade mucinous tumour was found, and abdominal dissemination was considered. **(D)** Immunohistochemistry: CK20(+),CDX-2(+),P53(wild type),Ki67 approximately 60%,CK7(-),MUC-2(+). It is a low-grade mucinous tumour.

After excluding surgical contraindications, the operation proceeded with general anesthesia and endotracheal intubation, followed by the removal of the right mesocolon, right inguinal region, and right femoral head tumours, and exploration revealed that the tumour body was located in the posterior right kidney, the midline of the psoas muscle, and that the tumour connected to the end of the appendix. The tumour’s membrane was intact, with a lower extension through the inguinal canal into the abdominal cavity, and a lower extension to the right femoral head. The abdominal cavity tumour had intact membrane, with full dissection of the tumour, followed by the ligation of the appendix artery and the appendix root (**Figure 2**). However, due to the adhesion between the tumour and the inguinal canal and the inferior gluteal artery, the tumour could not be completely separated, and the tumour was cut, revealing a yellow gelatinous substance inside. During the removal of the tumour in the right iliopsoas region, the tumour membrane was found to be tightly adhered to the anterior fascia lata and obliquus externus abdominis, with rupture during separation. The yellow gelatinous substance was released. During the removal of the tumour in the right femoral head, the tumour was found to be surrounded by the femoral nerve. To preserve the lower limb function, a palliative resection was performed. Postoperative specimens were sent for pathological examination, confirming the diagnosis of a low-grade appendix mucinous tumour with peritoneal mesothelioma (**Figure 3**).Due to the large size of the patient’s mass, it is tightly adhered to the femoral canal, inguinal artery and vein, and femoral nerve, making it impossible to remove the mass entirely. During the patient’s hospitalisation, the plan was to perform intraperitoneal hyperthermic perfusion chemotherapy. However, the patient and her family refused. One month after the surgery, the patient fully recovered and the surgical wound healed well. Follow-up visits were scheduled.

## Discussion

Histologically, a LAMN can cause atrophy or fibrosis of the lamina propria and advance into the appendiceal wall to produce a low-grade mucinous tumour epithelium with expansive or diverticulum-like growth (2). When the epithelium of a low-grade mucinous tumour invades the appendiceal wall to form the most dreaded complication of LAMA, PMP, which is characterised by mucinous ascites and peritoneal implants, and usually originates from a low-grade malignant mucinous tumour with a perforated appendix (3).In this case, the peritoneal pseudomucinous tumour in the patient may have arisen as a result of appendicular mucus penetrating the wall of the appendix and spreading into the peritoneal cavity in the form of a gelatinous deposit, growing along the posterior peritoneum, while the mass extended downwards to the lower part of the inguinal area to adhere tightly to the femoral canal and form a large tumour in the right iliopsoas muscle-right quadriceps muscle. Patients with peritoneal pseudomucinous tumours tend to have a painless clinical course, slow and persistent intraperitoneal growth of the tumour, low malignancy, and no typical metastatic spread (extraperitoneal). Usually, PMP exhibits the characteristic “jelly belly”, as the amount of mucoid fluid fills the peritoneal cavity (4).. Little is known about PMPs at this time. PMP is known to develop insidiously as a result of mucin-producing, neoplastic epithelial cup cells forming mucin implants throughout the abdominopelvic peritoneum; PMP usually recurs after surgical resection and is associated with significant morbidity and mortality (5).The patient in this case was found to have an inguinal mass 30 years ago, but did not pay attention to this mass. Since the onset of the disease, the patient has not experienced any discomfort, however, due to the continuous growth of the tumour, the tumour penetrated through the femoral canal and grew to the root of the thigh, and the compression of the femoral nerve caused pain in the thigh that prevented the patient from walking. He then came to the clinic for medical treatment.

Simple low-grade appendiceal mucinous tumours are usually treated with simple appendectomy and have a relatively low rate of postoperative recurrence; if the tumour ruptures and spreads to the peritoneum and viscera, forming a gel-like substance that results in the condition known as peritoneal pseudomucocele, the morbidity and mortality rates will be significantly higher (6). Thus, the ultimate goal in the management of such cases is to avoid appendiceal mucinous tumour rupture and peritoneal pseudomucinous tumour syndrome. Complete surgical resection of the tumour is the only possible cure. Acceptable treatments include appendectomy, right hemicolectomy, partial colectomy combined with tumour reduction or palliative resection combined with chemotherapy (7). Combined postoperative hyperthermic intraperitoneal perfusion chemotherapy (HIPEC) may prolong the survival of patients with these diseases (8). The 5-year survival rate for patients with low-grade appendiceal mucinous tumours is statistically 62.5%-100% when patients are treated with a combination of cytoreductive surgery (CRS) and hyperthermic intraperitoneal chemotherapy (HIPEC) (5). In this case, we resected the appendix and periappendiceal mass, resected the right iliac lumbar abdominal wall mass, and resected the skeletal muscle soft tissue tumour in the right inguinal region below and above the femur under open general anaesthesia. Because part of the mass in the right inguinal region below and above the femur was surrounded by the peripheral membrane of the nerve root, complete resection was not possible. Therefore, the mass was removed palliatively, and it is now believed that appendiceal mucinous tumours are susceptible to pseudoperitoneal mucocele. It was recommended that the patient and his family receive peritoneal hot perfusion chemotherapy complemented with medications for mucus lysis after surgery, however, his family refused to do so, and in addition, patients with mucous tumours in the appendix have a risk for rectal cancer that is six times higher than that of the general population; therefore, they should be followed up closely during the postoperative period.

In summary, low-grade appendiceal mucinous tumour are rare. The clinical presentation is often nonspecific, and clinicians should perform an adequate preoperative evaluation of the patient’s history, physical examination, and imaging to remove the mass as completely as possible and avoid rupture of the mucinous cyst, which may lead to peritoneal pseudomucinous tumours, a disease with high morbidity and mortality. Surgical access and procedures should be carefully chosen. If complete removal of the mass is not possible, palliative resection followed by combination chemotherapy may prolong patient survival.

## Data availability statement

The original contributions presented in the study are included in the article/supplementary material. Further inquiries can be directed to the corresponding author.

## Ethics statement

Written informed consent was obtained from the individual(s) for the publication of any potentially identifiable images or data included in this article. Written informed consent was obtained from the participant/patient(s) for the publication of this case report.

## Author contributions

YK: Writing – original draft. JM: Writing – review & editing. XL: Data curation, Supervision, Writing – review & editing. ZY: Conceptualization, Investigation, Writing – review & editing. MD: Writing – review & editing.
